# Association of mental health related quality of life and other factors with treatment seeking for substance use disorders: A comparison of SUDs rooted in legal, partially legal, and illegal substances

**DOI:** 10.1371/journal.pone.0302544

**Published:** 2024-04-29

**Authors:** John L. Havlik, Taeho G. Rhee, Robert A. Rosenheck

**Affiliations:** 1 Department of Psychiatry, Yale University School of Medicine, New Haven, Connecticut, United States of America; 2 Department of Public Health Sciences, University of Connecticut School of Medicine, Farmington, Connecticut, United States of America; 3 Department of Veterans Affairs Connecticut Healthcare System, West Haven, Connecticut, United States of America; University of Connecticut Health Center: UConn Health, UNITED STATES

## Abstract

The association of subjective mental health-related quality of life (MHRQOL) and treatment use among people experiencing common substance use disorders (SUDs) is not known. Furthermore, the association of a given substance’s legal status with treatment use has not been studied. This work aims determine the association of MHRQOL with SUD treatment use, and how substance legal status modulates this relationship. Our analysis used nationally-representative data from the NESARC-III database of those experiencing past-year SUDs (n = 5,808) to compare rates of treatment use and its correlates among three groups: those with illicit substance use disorders (ISUDs); those with partially legal substance use disorders, i.e., cannabis use disorder (CUD); and those with fully legal substance use disorders, i.e., alcohol use disorder (AUD). Survey-weighted multiple regression analysis was used to assess the association of MHRQOL with likelihood of treatment use among these three groups, both unadjusted and adjusted for sociodemographic, behavioral, and diagnostic factors. Adults with past-year ISUDs were significantly more likely to use treatment than those with CUD and AUD. Among those with ISUDs, MHRQOL had no significant association with likelihood of treatment use. Those with past-year CUD saw significant negative association of MHRQOL with treatment use in unadjusted analysis, but not after controlling for diagnostic and other behavioral health factors. Those with past-year AUD had significant negative association of MHRQOL with treatment use in both unadjusted and adjusted analysis. If legalization and decriminalization continue, there may be a greater need for effective public education and harm reduction services to address this changing SUD landscape.

## Introduction

With progressive decriminalization and legalization of cannabis within the United States, and potential near-term FDA approval of treatment with potentially therapeutic psychedelic substances, there appears to be a general trend toward legalization and decriminalization of many previously illicit substances [1,2]. Use of these substances is not without consequences. On the positive side, decriminalization (where use of previously illegal substances remains illegal, but the legal system does not prosecute these crimes) and legalization (where previously illicit substance use is no longer a crime) have been heralded as a way to advance health equity while reducing stigma associated with treatment seeking [3–6]. On the other hand, societal costs of substance use disorders (SUDs), related health care expenditures, law enforcement expenditures, and indirect costs such as unemployment have been estimated at nearly 6% of US gross domestic product annually, more than the costs of common medical conditions such as heart disease, stroke, and obesity, though the economic and social effects of legalization and decriminalization are not known [7–11]. With such high prevalence and costs of illness, determining rates and correlates of treatment seeking is of interest to the medical community, especially examining the association of the comparative legal status of various substances with rates and correlates of treatment use, an issue that has been little studied [12,13].

Several studies have been devoted to describing the impacts of legal status on the extent of substance use, primarily in association with progressive decriminalization and legalization of cannabis in the United States [14–17]. This literature is mixed on the question of whether and how cannabis legalization has impacted rates of cannabis use and cannabis use disorder (CUD), as well as rates of treatment use. Some studies suggest legalization is associated with increased cannabis use but not treatment seeking for CUD [18–23]. Concerningly, multiple studies using national databases have found that cannabis legalization has been associated with increased use of remaining illicit substances, suggesting general illicit substance use may increase as another substance is legalized [24,25].

Several studies have reported high rates of medical and psychiatric comorbidities in various SUDs [26–31]. While the prevalence of some of these comorbidities are well-established in the literature, there is limited data comparing sociodemographic, co-morbid diagnostic and Health Related Quality of Life (HRQOL) among those seeking treatment for substances with different legal statuses, i.e., totally legal (e.g., alcohol), partially legal with variations across states in legality and enforcement (cannabis), and universally illegal (e.g., heroin, amphetamines, illegally sold prescription drugs). Some recent data has provided a limited characterization of treatment use by legal status of the involved substances, finding adults with SUDs related to illegal substances such as heroin were both more likely to need and to receive treatment than those with the entirely legal AUD [32]. Still, it remains unclear whether differences in the legal status of various substances are associated with differences in comorbidities and personal experiences as reflected in Mental HRQOL (MHRQOL), a standardized measure of quality of life attributable to one’s mental health. Importantly, differences in MHRQOL may be associated with differences in the likelihood of use of SUD treatment, as our group has note in previous work [33].

In this study we use survey data from the National Epidemiologic Survey on Alcohol and Related Conditions (NESARC)–III to compare concurrent characteristics and services use among three distinct groups of SUDs defined by the legal status of the substances involved: legal, partially legal, and illegal. Our specific focus is on differences in subjective MHRQOL and its independent association with the likelihood of receiving treatment, as explored in a recent study of AUD based on NESARC-III [34]. We consider the following questions: 1) What are the significant differences in sociodemographic and clinical characteristics between diagnostically-defined groups that differ in the legal status of the substances at the root of their SUDs, 2) Are there differences in rates and correlates of treatment use between SUDs that differ in their legal statuses, including in the association with MHRQOL? And 3) To what extent are differences in subjective MHRQOL, reflecting personal subjective distress, associated with differences in receipt of SUD treatment for substances of differing legal status, both in unadjusted analyses and net of socio-demographic characteristics, co-morbid mental and medical disorders, and behavioral characteristics that are related to both MHRQOL and SUDs and thus are potential confounders? Through this investigation we seek to evaluate the independent role of subjective MHRQOL on use of services for SUDs with different legal statuses, differences that may be important if recent trends towards legalization and decriminalization continue.

## Materials and methods

### Data source and study sample

We used restricted data from the National Epidemiologic Survey on Alcohol and Related Conditions-III (NESARC-III), sponsored by the National Institute on Alcohol Abuse and Alcoholism (NIAAA) [35]. The NESARC-III is a nationally representative cross-sectional survey, conducted from April 2012 through June 2013, of physical and mental health diagnoses, well-being, and disabilities among non-institutionalized civilian adults aged 18 or older with a focus on alcohol and other SUDs. Participants in the NESARC survey gave electronically documented oral informed consent in a manner approved by the National Institutes of Health institutional review board [36]. In NESARC-III, the Alcohol Use Disorder and Associated Disabilities Interview Schedule-5 (AUDADIS-5) was used to identify psychiatric disorders using Diagnostic and Statistical Manual of Mental Disorders, 5th edition (DSM-5) criteria [37].

In this study, we included the entire sample of adults aged 18 or older who met criteria for any SUD (n = 5,808 unweighted). We classified the sample into three groups, stratified by substance legality, with no overlap between groups. One “legal” use disorder group was defined by inclusion of adults with past year AUD (n = 4,321), the only entirely legal substance for adults considered in our classification, as nicotine use disorder was not used to define this group. The next “partially legal” group was defined as including adults with past year CUD (n = 828), a substance that is legal in the form of both medical and recreational marijuana use with considerable variability across states. The final, “illicit” group was defined as including adults with past year illicit SUDs (ISUDs (n = 659), including cocaine use disorder, heroin use disorder, amphetamine or stimulant use disorder, hallucinogen use disorder, club drug use disorder, prescription opioid use disorder, sedative use disorder, or other unspecified drug use disorder. Adults who met criteria for more than one group were classified in the more illegal group (e.g., a person with AUD *and* CUD was placed in the “partially legal” group).

### Measures

#### Mental Health-Related Quality of Life (MHRQOL)

MHRQOL is a patient-reported indicator of health status and assesses subjective evaluation of the impact of disease on bio-psycho-social wellbeing from a mental health perspective [38]. Developed as part of the Medical Outcomes Study (MOS), the 12-item Short Form (SF-12) survey is a standardized questionnaire asking patients about their health states such as physical functioning, social functioning, role limitations, emotions, and general health [39]. Based on these 12 items, we constructed the mental component summary variable (MCS), a standardized measure of MHRQOL, using standard scoring algorithms that score the national average values on these measures as 50 and each standard deviation as a difference of 10 points above or below this average [40,41].

#### SUD treatment use

Treatment use for our cohort was defined as receiving potentially definitive treatment for any SUD. This measure addressed receipt of outpatient or residential treatments that would potentially lead to an improved likelihood of abstinence or clinically meaningful reduction in use. These services included treatment at a family services agency, outpatient clinic including outreach and day or partial programs, private physician, psychiatrist, psychologist, social worker, rehabilitation program, or other professional agency in the past year. They did not include crisis intervention, emergency room (ER) services, or inpatient hospitalization as these services offer time-limited assistance and not ongoing, potentially definitive treatment for SUDs.

#### Sociodemographic variables

Sociodemographic variables included: age, sex, race/ethnicity, marital status, employment (i.e., a job or business, either full-time or part-time including unpaid work), family income, education, and health insurance [42–44]. Urban vs. rural residence was also documented.

#### Psychiatric and substance use disorders

Past-year psychiatric disorder diagnoses available in NESARC-III based on the AUDADIS and DSM 5 criteria [45] include major depressive disorder (hierarchical), dysthymia (hierarchical), bipolar I disorder, generalized anxiety disorder, post-traumatic stress disorder, eating disorders, specific phobias, and panic disorder. Hierarchical disorders included specific disorders also meeting criteria for more general DSM-5 diagnoses, e.g., all in sample with listed major depressive disorder (hierarchical) also met criteria for the more general diagnosis of major depressive episode. We further constructed a summary three-level variable representing number of psychiatric disorders in the past year: none, one, or two or more, in the interest of parsimonious analysis.

#### Behavioral and other diagnostic factors

Behavioral and diagnostic factors accounted for in NESARC-III were selected for inclusion in our model based upon hypothesis-driven association with legality of substances used. Behavioral factors assessed in our study sample included MHRQOL, number of lifetime violent experiences, total 2-week contacts, perceived social support (assessed using the Interpersonal Support Evaluation List-12 (ISEL-12) [46], trouble with police in the past year, veteran status, and combat experience. Diagnostic variables of interest included chronic pain and medical comorbidities. Medical comorbidities were assessed as a summary three-level variable representing number of comorbid medical disorders: none, one, or two or more, in the interest of parsimonious analysis.

### Analysis

First, we investigated differences in sociodemographic, behavioral, and diagnostic characteristics for our three categories of SUDs (ISUD, CUD, and AUD), through three sets of pairwise comparisons (ISUD vs CUD, CUD vs AUD, and ISUD vs AUD). Comparisons of categorical variables were based on bivariable logistic regression analyses with odds ratios as indicators of effect sizes while continuous variables were compared with Cohen’s d and associated P-values as indicators of statistical significance and effect size differences. P-values were assessed as significant if <0.05.

Next, after identifying significant differences in these characteristics among our three groups, we examined differences in rates of treatment use within each of the groups, through 1) both unadjusted pairwise bivariable odds ratios comparisons and 2) with adjustment for all significantly different sociodemographic, behavioral, and diagnostic characteristics identified above.

Third, we subsequently quantified differences in sociodemographic, behavioral, and diagnostic characteristics between treatment users and non-users within each SUD group using logistic regression and Cohen’s d as described above.

Finally, we investigated the independent association of MHRQOL and likelihood of SUD treatment within each of our three SUD groups. We constructed three sets of models for each group. The first model was a bivariable logistic regression assessing the unadjusted association MHRQOL with likelihood of SUD treatment. The second model added covariates representing sociodemographic variables such as age, sex, and race that may confound this association. The third model added additional covariates representing behavioral factors such as trouble with police in the past year and diagnostic factors like number of diagnosed non-SUD psychiatric disorders.

As we aimed to control for three sets of factors in nine multivariable analyses, we undertook a Bonferroni correction for multiple comparisons by considering test results significant at p<0.05/9 = 0.005. We used Stata version 17.0 for all analyses and employed the *svy* commands in Stata to account for the complex survey sampling design of the NESARC-III (e.g., unequal probability of selection, clustering and stratification) [47].

The study procedures were approved by the Institutional Review Board (#2000022543) at Yale School of Medicine. Further details of the survey, including descriptions, questionnaires, sampling methodology and datasets, are available on the NESARC- III website [35].

## Results

### Sample

Overall, 16.0% (n = 5,808) of the entire study sample, representative of 36,887,003 adults, met criteria for an included SUD within the past 12 months: 659 (11.4%) with ISUDs, representative of 4.1 million adults, 828 (14.3%) with CUD, representative of 5.1 million adults, and 4,321 with AUD (74.4%), representative of 27.7 million adults (Table 1, rows 2–3).

**Table 1:** Socio-demographic, behavioral, and medical characteristics (weighted column %) of US adults by substance use disorder status, NESARC-III.

	Current ISUD	Current CUD	Current AUD	OR/Cohen’s D ISUD vs CUD	OR/Cohen’s D CUD vs AUD	OR/Cohen’s D ISUD vs AUD	Paired comparisons based on effect size
**Sample size**							
Unweighted sample (row %)	659 (11.4)	828 (14.3)	4,321 (74.4)	0.80	0.19	0.15	
Weighted population (% total population)	4,064,620 (1.8)	5,066,631 (2.3)	27,755,752 (11.9)	1.76	0.29	0.51	
**Age**							
18–44	61.7	84.3	70.0	0.30***	2.30***	0.69**	
45–64	32.7	13.9	26.6	3.01***	0.44***	1.34*	1,3>2
65+	5.6	1.8	3.4	3.28**	0.52	1.72*	1>3>2
**Female**	48.4	32.8	39.4	1.92***	0.75**	1.44***	1>2
**Race/ethnicity**							
Non-Hispanic white	71.0	55.9	67.8	1.93***	0.60***	1.17	
Non-Hispanic black	13.2	21.6	11.0	0.55***	2.23***	1.22	2>1,3
Hispanic	13.0	16.0	14.4	0.79	1.14	0.89	
Other	2.8	6.5	6.9	0.42*	0.93	0.39**	1< 2,3
**Unmarried**	75.2	81.9	65.6	0.67*	2.37***	1.59***	
**Family income**							
<$20,000	51.7	48.4	30.0	1.14	2.19***	2.50***	1,2>3
$20,000 –$39,999	17.4	18.7	17.7	0.92	1.06	0.98	
≥$40,000	30.9	33.0	52.3	0.91	0.45***	0.41***	1,2<3
**Employed**	66.4	77.6	84.3	0.57***	0.64***	0.37***	
**Education**							
<High school	25.3	23.0	13.8	1.13	1.87***	2.12***	1,2>3
High school or equivalent	43.1	45.4	35.6	0.91	1.50***	1.37*	
Some college	12.8	11.6	14.0	1.11	0.81	0.9	
≥Bachelor’s degree	18.9	20.0	36.6	0.93	0.43***	0.40***	1,2<3
**Insurance coverage**							
Medicare (%)	7.3	3.7	2.9	2.06*	1.28	2.64***	1>2,3
Medicaid (%)	16.9	15.9	7.8	1.08	2.23***	2.41***	1,2>3
Private (%)	39.1	40.5	57.0	0.94	0.51***	0.48***	
Other (%)	10.8	12.0	10.0	0.88	1.23	1.09	
**Urban residence**	82.5	84.2	83.8	0.88	1.03	0.91	
**Past-year psychiatric disorders**							
0	23.1	38.2	57.4	0.49***	0.46***	0.22***	1<2<3
1	19.0	18.8	18.7	1.01	1.00	1.02	
2+	58.0	43.0	23.9	1.83***	2.40***	4.39***	1,2 >3
**Past-year substance use disorders**							
Alcohol	49.4	56.9	-	-	-	-	
Cannabis	22.5	-	-	-	-	-	
Other Illicit	-	-	-	-	-	-	
**Behavioral Characteristics**							
Mental Health Related QOL (Mean ± SD)	40.6 ± 11.9	46.1 ± 11.1	48.4 ± 10.1	-0.46***	-0.22***	-0.73***	1<2<3
Violent experiences (Mean ± SD)	1.7 ± 1.8	1.4 ± 1.5	1.0 ± 1.3	0.30***	0.52***	0.20**	1>2>3
Total 2-week contacts (Mean ± SD)	13.6 ± 13.7	15.2 ± 14.2	17.0 ± 16.4	-0.12	-0.11*	-0.21***	1<3
Social support (Mean ± SD)	2.8 ± 0.6	2.9 ± 0.5	3.0 ± 0.5	-0.16**	-0.27***	-0.44***	1,2 <3
Trouble with police in past year (%)	10.7	9.8	4.1	1.09	2.39***	2.61***	1,2 >3
Veteran (%)	8.0	7.7	8.3	1.04	0.93	0.96	
Combat experience (%)	1.9	3.0	2.8	0.63	1.07	0.68	
Chronic pain (%)	44.9	21.5	16.8	2.09***	1.28**	2.67***	1>2,3
**Medical comorbidities (%)**							
0	41.0	61.7	65.1	0.43***	0.87	0.37***	1<2,3
1	27.1	20.9	20.6	1.41*	1.01	1.43**	
2+	31.9	17.4	14.3	2.22***	1.27*	2.82***	1>2,3

*: P < 0.05

**: P < 0.01

***: P < 0.001. ISUD: Illicit substance use disorder; CUD: Cannabis use disorder; AUD: Alcohol use disorder; OR: Odds ratio; QOL: Quality of life; SD: Standard deviation. If more than one drug category is present the observation is included is the "more illegal" category (IUD>CUD>AUD).

### SUD legality-stratified differences in background characteristics and comorbidities

There were significant differences between pairs of substance groups on many sociodemographic, psychiatric, medical, and behavioral measures (Table 1). The group with past year ISUD (whether treated or not) was older, more likely to be male, less likely to be employed, and had lower incomes than those who met criteria for CUD or AUD. The group with ISUDs had a significantly greater risk of psychiatric disorders compared to those with CUD or AUD. The group also had a higher number of lifetime violent experiences, lower amount of self-assessed social support, and higher likelihood of experiencing chronic pain than those with CUD or AUD. This group with ISUD had a higher number of medical comorbidities than those with CUD or AUD. Notably, the group with ISUD had significantly lower MHRQOL as reflected by their MCS scores (40.6 ± 11.9) than those with CUD (46.1 ± 11.1) and AUD (48.4 ± 10.1) (Table 1).

Examining other differences among groups, those with CUD were more likely to be younger than those with ISUD or AUD, were less likely to be white than those with ISUD or AUD, and were more likely to be non-Hispanic black (Table 1). While they did not have as great a risk for past-year psychiatric disorders as those with ISUD, they had significantly greater risk of these disorders than those with AUD (Table 1).

Those with AUD were most likely to be married (34.4%), had higher incomes, were more likely to be employed, have higher levels of education, and have fewer psychiatric disorders than the other two groups.

### Differences in rates of treatment by substance legality

Overall, 627 patients (10.7%), representing 3.8 million adults, were treated for SUDs in the past 12 months, while 5,181 patients (89.2%), representing 33.1 million adults, did not receive treatment. Treatment rates varied significantly by legality class of substances of use (Table 2): 26.9% of those with past-year ISUDs, representing 1.1 million US adults, received SUD treatment, while just 10.3% of those with past-year CUD (representing 612,389 US adults) and 8.5% of those with past-year AUD (representing 2.1 million US adults) received treatment. Those with ISUDs had a significantly higher likelihood of receiving treatment than those with CUD (Odds ratio: 2.62, P<0.001) or AUD (Odds ratio: 4.35, P<0.001); these differences persisted after adjustment for sociodemographic, behavioral, and diagnostic characteristics. Those with CUD had significantly higher likelihood of receiving treatment than those with AUD (Odds ratio: 1.66, P<0.01), a difference that was no longer statistically significant after adjusting for sociodemographic, behavioral, and diagnostic characteristics.

**Table 2:** Comparison of current substance use among US adults stratified by legality-based substance use disorder type.

	ISUD, No. (%)	CUD, No. (%)	AUD, No. (%)	Odds of treatment use, ISUD vs CUD, OR (aOR)	Odds of treatment use, CUD vs AUD, OR (aOR)	Odds of treatment use, ISUD vs AUD, OR (aOR)
Treatment Users	177 (26.9)	85 (10.3)	365 (8.5)	2.62*** (2.60***)	1.66** (1.14)	4.35*** (2.62***)
Treatment Non-users	482 (73.1)	743 (89.7)	3,956 (91.6)	**-**	**-**	**-**

*:P < 0.05

**: P < 0.01

***: P < 0.001. ISUD: Illicit substance use disorder; CUD: Cannabis use disorder; AUD: Alcohol use disorder; OR: Odds ratio, aOR adjusted odds ratio (adjusted for all significantly different sociodemographic, behavioral, and diagnostic characteristics in Table 1).

### Correlates of SUD treatment use

Within legality-based groups of substances, treatment status was associated with significant differences in diverse characteristics, diagnoses, and comorbidities (Table 3). Stratified by age, those with ISUDs, receiving SUD treatment were less likely to be 65+; those receiving treatment for AUD were more likely to be 45–64 and less likely to be 18–44. Those with AUD receiving SUD treatment were significantly more likely to be unmarried. Stratifying by family income, those receiving treatment for AUD were more likely than others with AUD to have an income of <$20,000 per year and less likely to have an income of over $40,000 per year. Those with AUD who received treatment were also less likely to be employed, and more likely to have completed less than a high school education than others with AUD.

**Table 3:** Comparison of treated and untreated adults, classified by the legality of substance use, and compared with statistical comparison using odds ratios and Cohen’s D.

	Current ISUD treated	Current ISUD untreated	Current CUD treated	Current CUD untreated	Current AUD treated	Current AUD untreated	OR/Cohen’s D Treated vs Untreated ISUD	OR/Cohen’s D Treated vs Untreated CUD	OR/Cohen’s D Treated vs Untreated AUD
**Sample size**									
Unweighted sample (% sample)	177 (26.9)	482 (73.1)	85 (10.3)	743 (89.7)	365 (8.5)	3,956 (91.6)	0.37	0.11	0.09
Weighted population (% total population)	1,076,019 (0.5)	2,988,601 (1.3)	612,389 (0.2)	4,454,243 (2.1)	2,120,859 (1.0)	25,634,892 (10.9)	0.36	0.14	0.08
**Age**									
18–44	64.9	60.5	80.4	84.9	61.7	70.7	1.21	0.73	0.67**
45–64	34.0	32.2	17.8	13.4	35.9	25.8	1.08	1.41	1.61***
65+	1.1	7.3	1.8	1.8	2.4	3.4	0.14*	1.00	0.69
**Female**	45.9	49.3	37.8	32.1	40.3	39.3	0.87	1.29	1.04
**Race/ethnicity**									
Non-Hispanic white	74.5	69.7	53.9	56.2	69.8	67.6	1.27	0.91	1.11
Non-Hispanic black	10.8	14.0	17.7	22.2	11.6	11.0	0.74	0.75	1.06
Hispanic	13.3	12.9	12.0	16.5	12.0	14.6	1.04	0.69	0.80
Other	1.4	3.3	16.5	5.1	6.7	6.9	0.40	3.70*	0.96
**Unmarried**	80.2	73.4	87.1	81.2	73.7	64.9	1.46	1.56	1.52*
**Family income**									
<$20,000	54.6	50.7	61.6	46.5	41.9	29.0	1.17	1.84	1.77***
$20,000 –$39,999	15.7	18.0	11.4	19.7	17.7	17.7	0.85	0.53	1.00
≥$40,000	29.8	31.4	27.1	33.8	53.3	40.3	0.93	0.73	0.59***
**Employed**	63.3	67.6	70.8	78.5	77.4	84.9	0.83	0.66	0.61***
**Education**									
<High school	20.1	27.1	14.8	24.1	20.9	13.2	0.67	0.55	1.74**
High school or equivalent	45.2	42.4	48.8	44.9	31.9	35.9	1.12	1.17	0.83
Some college	16.9	11.3	19.7	10.6	17.9	13.7	1.60	2.07	1.37
≥Bachelor’s degree	17.9	19.2	16.8	20.4	29.4	37.2	0.91	0.78	0.70
**Insurance coverage**									
Medicare (%)	6.7	7.5	0.7	4.1	3.1	2.9	0.89	0.17*	1.08
Medicaid (%)	19.5	16.0	16.2	15.8	18.7	6.9	1.27	1.03	3.10***
Private (%)	39.1	39.1	28.7	42.1	43.7	58.1	1.00	0.55*	0.56***
Other (%)	9.2	11.4	29.4	9.6	7.2	10.2	0.79	3.91***	0.68
**Urban residence**	84.9	81.6	90.0	83.4	86.5	83.6	1.27	1.78	1.25
**Past-year psychiatric disorders**									
0	18.6	24.7	16.7	41.2	32.7	59.4	0.70	0.29***	0.33***
1	13.2	21.1	18.9	18.8	18.8	18.7	0.57*	1.01	1.00
2+	68.2	54.3	64.4	40.1	48.5	21.9	1.80*	2.70***	3.37***
**Past-year substance use disorders**									
Alcohol	61.3	45.1	75.8	54.4	-	-	1.93**	2.64*	-
Cannabis	28.5	20.4	-	-	-	-	1.55*	-	-
Other Illicit	-	-	-	-	-	-	-	-	-
**Behavioral Characteristics**									
Mental health related QOL (Mean ± SD)	38.8 ± 12.6	41.3 ± 11.7	41.1 ± 11.4	46.8 ± 11.0	42.6 ± 12.6	48.9 ± 9.7	-0.21	-0.51***	-0.62***
Violent experiences (Mean ± SD)	2.1 ± 2.0	1.6 ± 1.8	1.9 ± 1.7	1.3 ± 1.4	1.5 ± 1.5	1.0 ± 1.3	0.28	0.40**	0.38***
Total 2-week contacts (Mean ± SD)	12.5 ± 11.8	13.9 ± 14.3	15.6 ± 14.5	15.2 ± 14.2	17.4 ± 22.8	16.9 ± 15.7	-0.10	0.03	0.03
Social support (Mean ± SD)	2.7 ± 0.6	2.8 ± 0.6	2.7 ± 0.6	2.9 ± 0.5	2.8 ± 0.6	3.0 ± 0.5	-0.17	-0.40*	-0.40
Trouble with police in past year (%)	14.8	9.3	25.0	7.7	10.1	3.7	1.69	3.97***	2.97***
Veteran (%)	8.3	8.0	18.6	6.2	10.0	8.2	1.04	3.48**	1.25
Combat experience (%)	3.2	1.4	7.2	2.4	3.8	2.8	2.36	3.14	1.41
Chronic pain (%)	41.2	46.2	23.9	21.2	28.7	15.8	0.82	1.17	2.15***
**Medical comorbidities (%)**									
0	45.5	39.4	55.0	62.7	53.0	66.1	1.28	0.73	0.58***
1	21.6	29.0	14.1	21.8	25.9	20.2	0.68	0.59	1.38
2+	32.9	31.6	30.9	15.6	21.1	13.7	1.06	2.42*	1.69**

* P<0.05.

**P<0.01

***P<0.001. ISUD: Illicit substance use disorder; CUD: Cannabis use disorder; AUD: Alcohol use disorder; OR: Odds ratio; QOL: Quality of life; SD: Standard deviation.

Examining diagnostic and behavioral variables, those receiving treatment for SUDs across all groups tended to have more than one psychiatric disorder compared to those not receiving treatment. They also tended to have higher rates of comorbid substance use.

Notably, MHRQOL was not significantly different among those with ISUDs receiving and not receiving SUD treatment. Among those with CUD, those receiving SUD treatment had significantly lower MHRQOL (Cohen’s d -0.51, P<0.001), were more likely to report trouble with police in the past year, and were more likely to be veterans than those not receiving treatment.

As in those with CUD, those with AUD receiving SUD treatment had significantly lower MHRQOL (Cohen’s d -0.62, P<0.001) than those not receiving SUD treatment. Those receiving treatment for CUD and AUD, but not ISUD, as compared to those not receiving treatment, had a significantly higher likelihood of reporting a history of violent experiences, and to report trouble with the police in the past year. Those with CUD who received treatment were more likely to be veterans, and reported significantly less social support than those who did not receive treatment. Those who received treatment for AUD were more likely to report chronic pain and to have 2 or more medical comorbidities than those not receiving treatment for AUD.

### Multivariate group comparisons on measures of MHRQOL

Multivariable-adjusted comparisons within legality-based groups of likelihood of seeking treatment focused on the independent contribution (or lack thereof) of MHRQOL to the likelihood of seeking treatment net of other factors. Unadjusted regression coefficients are presented in columns 2, 5 and 8 of Table 4 and reflect differences in MHRQOL between in treated and non-treated subgroups within each legality group. In unadjusted bivariable logistic regression, higher MHRQOL is significantly and negatively associated with likelihood of treatment in those with CUD (-4.1% for each additional point of MCS) and AUD (-5.0% for each additional point of MCS), but there was no significant association among those with ISUD (Table 4, Columns 2, 5 and 8). These associations between MHRQOL and likelihood of treatment persist, though slightly attenuated, after adjusting for sociodemographic differences, (Table 4, Columns 6 and 9). After further adjusting for mental health diagnoses and behavioral indicators, higher MHRQOL remains a significant negative correlate of treatment seeking only for the AUD group (Table 4, Column 10). In this final set of models for the AUD group, each additional point in the MCS score was associated with 3.6% lower odds of receiving SUD treatment.

**Table 4:** Multivariable analyses of likelihood of receiving treatment quality of life (QOL) and among US adults with SUDs, stratified by substance use disorder legality †.

	ISUD			CUD			AUD		
	Bivariate OR	Adjusted for Demograp-hics OR	Adjusted for Diagnostic and Behavioral Data OR	Bivariate OR	Adjusted for Demograp-hics OR	Adjusted for Diagnostic and Behavioral Data OR	Bivariate OR	Adjusted for Demograp-hics OR	Adjusted for Diagnostic and Behavioral Data OR
**Mental Health Related QOL**	0.983	0.981	0.986	0.959*	0.961*	0.980	0.950*	0.954*	0.964*
**Age**									
18–44	REFERENCE			REFERENCE			REFERENCE		
45–64	-	0.936	0.945	-	1.056	0.785	-	1.578*	1.677
65+	-	0.151	0.154	-	1.683	0.822	-	0.758	0.843
**Female**	-	0.828	0.809	-	1.022	1.265	-	0.800	0.831
**Race/ethnicity**									
Non-Hispanic white	REFERENCE			REFERENCE			REFERENCE		
Non-Hispanic black	-	0.758	0.8	-	0.839	0.726	-	0.656	0.683
Hispanic	-	0.965	0.968	-	0.711	0.667	-	0.655	0.685
Other	-	0.369	0.346	-	2.278	2.157	-	0.859	0.727
**Unmarried**	-			-	-	-	-	1.437	1.300
**Family income**									
<$20,000	REFERENCE			REFERENCE			REFERENCE		
$20,000 –$39,999	-	-	-	-	-	-	-	0.995	1.124
≥$40,000	-	-	-	-	-	-	-	0.866	0.891
**Employed**	-	-	-	-	-	-	-	0.907	0.870
**Education**									
<High school	REFERENCE			REFERENCE			REFERENCE		
High school or equivalent	-	-	-	-	-	-	-	0.611	0.662
Some college	-	-	-	-	-	-	-	1.042	1.103
≥Bachelor’s degree	-	-	-	-	-	-	-	0.84	1.054
**Insurance coverage**									
Medicare (%)	REFERENCE			REFERENCE			REFERENCE		
Medicaid (%)	-	-	-	-	0.194	0.158	-	-	-
Private (%)	-	-	-	-	0.811	0.999	-	2.111	2.044
Other (%)	-	-	-	-	-	-	-	0.786	0.871
**Past-year psychiatric disorders**									
0	REFERENCE			REFERENCE			REFERENCE		
1	-	-	0.751	-	-	1.402	-	-	1.189
2+	-	-	1.428	-	-	1.946	-	-	2.032*
**Behavioral Characteristics**									
Violent experiences	-	-	-	-	-	1.209	-	-	1.169*
Social support	-	-	-	-	-	0.716	-	-	-
Trouble with police in past year (%)	-	-	-	-	-	4.268*	-	-	2.305
Veteran (%)	-	-	-	-	-	3.336	-	-	-
Combat experience (%)	-	-	-	-	-	1.019	-	-	-
Chronic pain (%)	-	-	-	-	-	-	-	-	1.094
**Medical comorbidities (%)**									
0	REFERENCE			REFERENCE			REFERENCE		
1	-	-	-	-	-	0.558	-	-	1.067
2+	-	-	-	-	-	1.346	-	-	0.811

† Inclusion criteria: Significance in bivariate logistic regression analysis in Table 3.

* P<0.005. ISUD: Illicit substance use disorder; CUD: Cannabis use disorder; AUD: Alcohol use disorder; OR: Odds ratio; QOL: Quality of life.

## Discussion

This study used data from a nationally representative sample of US adults to investigate the relationship between MHRQOL and SUD treatment use among individuals with current SUDs involving substances with different legal statuses. The results revealed, first, a significantly greater likelihood of treatment use among those with ISUDs as compared to partially legal CUD and fully legal AUD and second, significant differences in sociodemographic and clinical characteristics among groups classified by legal status of the substances underlying their SUDs. Third, the results revealed further differences between these groups in individual characteristics associated with treatment use, especially a significant association between lower MHRQOL and treatment use among individuals with the partially legal CUD and entirely legal AUD. In contrast, no significant association was observed between lower MHRQOL and treatment use among those with ISUDs. After adjusting for demographic factors, other mental health diagnoses, and behavioral indicators, the correlation of lower MHRQOL and greater treatment use remained significant for AUD but not for CUD, suggesting a more robust relationship between subjective distress and treatment use for SUDs based on fully legal substances than among partially legal or illegal SUDs.

This robust constellation of findings offers no clear interpretation. The divergent impact of substance legality on treatment use suggests that treatment use may be at least partially influenced by the legal status of the substances being used. One might have expected that this difference was driven by interactions with the police and legal system which might be expected to create pressure to obtain treatment among those with ISUDs, far more than for CUD or AUD. However, while reports of trouble with the police were more common among those with ISUDs, they had no significant relationship to treatment within this group. In contrast, contact with police, although less frequent, was significantly associated with treatment among those with CUD and AUD. It is possible that fear of negative interaction with the police or legal system, rather than actual interactions with police or the legal system, drive greater proportions of those with ISUDs to seek treatment. These fears would be expected to be lower among those with CUD and AUD and would likely be reduced among those with current ISUDs if patterns of legalization and decriminalization continue.

Surprisingly, while age greater than 65 and having more than one co-morbid psychiatric disorder were significantly associated with SUD treatment in bivariate analysis of the ISUD group, no specific risk factors were independently associated with treatment use among those with ISUDs on multivariate analysis, suggesting that amidst their many problems, what leads those with ISUDs to treatment is not a distinct set of individual characteristics measured in NESARC-III. It may be that treatment use reflects unmeasured factors other than individual characteristics, for example greater local availability of treatment facilities or some other unmeasured neighborhood characteristics. Proximity to substance use treatment, i.e., small area variations in service availability, that are not captured in NESARC-III beyond delineating residents as “urban” or “rural,” have been well characterized as factors associated with variations in treatment use in prior literature and may account for variation in use of treatment [48–50]. It is also possible that interaction effects of sex and MHRQOL-affecting comorbidities could have played a role, as women with opioid use disorder have been reported to exhibit more psychiatric comorbidities than men [51]. Another factor not captured in this analysis is the local availability of religious institutions that have programs to facilitate entry into SUD treatment that are also undocumented in NESARC-III [52,53].

In contrast to ISUDs, multiple individual factors were associated with treatment on multivariate analysis among those with CUD and AUD, especially low MHRQOL, and among those with AUD, multiple psychiatric co-morbidities. The significant association of low MHRQOL and treatment use among those with CUD and AUD suggests that for these disorders, subjective dissatisfaction with life plays an especially important role in treatment seeking for partially or fully legal SUDs, in contrast to ISUDs. Treatment appears to be more “elective” in those for whom legal substances are the basis for their SUD and thus more driven by subjective HRQOL.

Findings from this study have noteworthy policy implications. If legalization becomes more common, treatment rates for currently illicit substances may decrease and come to resemble those of currently legal or partially legal substances. Treatment use may also become more prominently influenced by public attitudes, which may be shaped by public service announcements and anti-drug information campaigns. Unfortunately, previous research has shown models of education such as D.A.R.E and most other public service messaging around curbing illicit substance use do not appear to be effective and more research is thus needed to develop effective public education interventions that encourage those suffering from legal SUDs to enter treatment and address their SUDs [54,55]. While overall quality of life for those with SUDs may improve as previously illicit substances become decriminalized (and fears of legal punishment are reduced), the risk of dying or other adverse health effects from excessive use of newly legalized substances, as is currently the case with alcohol, remains substantial and may be more effectively addressed by newly developed public information campaigns highlighting serious health risks [56].

Since improvements in substance-related MHRQOL due to legalization of previously illicit substances, could decrease rates of treatment use as people with ISUDs feel “good enough” to think they do not “need” treatment, new and more effective methods of reducing harm among those who choose not to seek treatment may also become increasingly important tools (50,51). Until public education campaigns improve their record of limited efficacy in encouraging treatment use, methods such as harm reduction may take on a greater role in caring for those with newly decriminalized SUDs, e.g., providing safe spaces for substance use where life-saving help in the event of overdose or other medical complications can be readily available [57,58].

Several limitations of this study deserve comment. First, the most recent available NESARC survey was conducted between 2012 and 2013, and substance use habits, attitudes and socio-economic conditions of the United States population have shifted since this time. However, data from this early period of decriminalization/legalization still may be informative for future developments and policy discussion. Among the major changes since 2013 are the legalization or decriminalization of recreational cannabis in many states, the vast increase in opioid overdose deaths due to the illegal prescribing and marketing of long-acting opiates such as oxycodone [59], and the growing availability of fentanyl and analogs in unstandardized and tragically often lethal doses [60,61]. Furthermore, we classified opioid use disorder in our “illegal” category, when many opioids are obtained legally and prescribed through physicians [62]. In addition, use of both legal and illegal substances increased during the COVID-19 pandemic [63–66]. Whether these changes would change the findings reported here is not known. Second, some underserved populations (e.g., homeless individuals and prisoners) were not surveyed in NESARC-III, and thus this study may underestimate the numbers of adults with all SUDs, but especially ISUDs which can result in incarceration. Here too, it is unclear whether inclusion of these respondents in NESARC-III would have changed our results. Third, the cross-sectional nature of this study meant the effects of receiving definitive and longitudinal treatment for SUDs on MHRQOL could not be assessed. Finally, consistent with other epidemiologic surveys, NESARC- III is not a clinician-administered interview yielding diagnoses but relies on the AUDADIS-5, a fully structured interview [45]. However, the reliability and validity of NESARC-III have been well documented in a number of studies [67–69].

## Conclusions

Despite these limitations, to our knowledge this is the first study to characterize the differences in proportions and correlates of adults receiving treatment for SUD by the legal status of the substances involved. More specifically, we have identified distinct differences in the strength of association of poor MHRQOL along with other factors, with treatment use, especially for partially legal and legal substances, which has not been previously noted. As legalization progresses, if indeed it does, there may be less latent legal pressure for treatment seeking and a greater need for effective methods of public education on the long-term adverse effects of SUDs to motivate use of treatment, as well as for harm reduction interventions for those who make hazardous, if legal, use of harmful substances. While legalization and decriminalization of previously illegal substances is unlikely to proceed rapidly in the near future, the analyses presented here may add useful information for discussions that will inevitably be controversial.

## Supporting information

S1 Checklist STROBE statement—checklist of items that should be included in reports of observational studies. (DOCX)
