# Supplementary material for: Association of mental health related quality of life and other factors with treatment seeking for substance use disorders: A comparison of SUDs rooted in legal, partially legal, and illegal substances
Source: PLoS One. 2024 Apr 29;19(4):e0302544. doi: 10.1371/journal.pone.0302544 (PMC11057773; doi:10.1371/journal.pone.0302544)
Supplement: S1 Checklist — (DOCX) [file pone.0302544.s001.docx]

STROBE Statement—checklist of items that should be included in reports of observational studies

|  | Item No. | Recommendation | Page  No. | Relevant text from manuscript |
| --- | --- | --- | --- | --- |
| **Title and abstract** | 1 | (*a*) Indicate the study’s design with a commonly used term in the title or the abstract | 1 | “Association of mental health related quality of life and other factors with treatment seeking for substance use disorders: A comparison of SUDs rooted in legal, partially legal, and illegal substances” |
|  |  | (*b*) Provide in the abstract an informative and balanced summary of what was done and what was found | 2 | “This analysis used nationally-representative data from the NESARC-III database of those experiencing past-year SUDs (n=5,808) to compare rates of service use and its correlates among three groups” |
| Introduction | | | |  |
| Background/rationale | 2 | Explain the scientific background and rationale for the investigation being reported | 3 | “determining rates and correlates of treatment seeking is of interest to the medical community, especially examining the association of the comparative legal status of various substances of abuse with rates and correlates of service use, an issue that has been little studied” |
| Objectives | 3 | State specific objectives, including any prespecified hypotheses | 4 | “We consider the following questions: 1) What are the significant differences in sociodemographic and clinical characteristics between diagnostically-defined groups that differ in the legal status of the substances at the root of their SUDs, 2) Are there differences in rates and correlates of service use between SUDs that differ in their legal statuses, including in the association with MHRQOL? And 3) To what extent are differences in subjective MHRQOL, reflecting personal subjective distress, associated with differences in receipt of SUD treatment for substances of differing legal status, both in unadjusted analyses and net of socio-demographic characteristics, co-morbid mental and medical disorders, and behavioral characteristics that are related to both MHRQOL and SUDs and thus are potential confounders?” |
| Methods | | | |  |
| Study design | 4 | Present key elements of study design early in the paper | 4 | “In this study we use survey data from the National Epidemiologic Survey on Alcohol and Related Conditions (NESARC) – III to compare concurrent characteristics and services use among three distinct groups of SUDs defined by the legal status of the substances involved: legal, partially legal, and illegal. Our specific focus is on differences in subjective MHRQOL and its independent association with the likelihood of receiving treatment” |
| Setting | 5 | Describe the setting, locations, and relevant dates, including periods of recruitment, exposure, follow-up, and data collection | 5 | “We used restricted data from the National Epidemiologic Survey on Alcohol and Related Conditions-III (NESARC-III), sponsored by the National Institute on Alcohol Abuse and Alcoholism (NIAAA) (33). The NESARC-III is a nationally representative cross-sectional survey, conducted from April 2012 through June 2013, of physical and mental health diagnoses, well-being, and disabilities among non-institutionalized civilian adults aged 18 or older with a focus on alcohol and other SUDs.” |
| Participants | 6 | (*a*) *Cohort study*—Give the eligibility criteria, and the sources and methods of selection of participants. Describe methods of follow-up  *Case-control study*—Give the eligibility criteria, and the sources and methods of case ascertainment and control selection. Give the rationale for the choice of cases and controls  *Cross-sectional study*—Give the eligibility criteria, and the sources and methods of selection of participants | 5 | “We used restricted data from the National Epidemiologic Survey on Alcohol and Related Conditions-III (NESARC-III), sponsored by the National Institute on Alcohol Abuse and Alcoholism (NIAAA) (33). The NESARC-III is a nationally representative cross-sectional survey, conducted from April 2012 through June 2013, of physical and mental health diagnoses, well-being, and disabilities among non-institutionalized civilian adults aged 18 or older with a focus on alcohol and other SUDs.” |
|  |  | (*b*) *Cohort study*—For matched studies, give matching criteria and number of exposed and unexposed  *Case-control study*—For matched studies, give matching criteria and the number of controls per case | 9 | “Overall, 16.0% (n= 5,808) of the entire study sample, representing 36,887,003 adults, met criteria for an included SUD within the past 12 months: 659 (11.4%) with ISUDs, representing 4.1 million adults, 828 (14.3%) with CUD, representing 5.1 million adults, and 4,321 with AUD (74.4%), representing 27.7 million adults (Table 1, rows 2-3).” |
| Variables | 7 | Clearly define all outcomes, exposures, predictors, potential confounders, and effect modifiers. Give diagnostic criteria, if applicable | 5 | “We classified the sample into three groups, stratified by substance legality, with no overlap between groups. One use disorder group was defined by inclusion of adults with past year AUD (n=4,321), the only entirely legal substance for adults considered in our classification, as nicotine use disorder was not used to define this group. The next “partially legal” group was defined as including adults with past year CUD (n=828), a substance that is legal in the form of both medical and recreational marijuana use with considerable variability across states. The final, “illicit” group was defined as including adults with past year illicit SUDs (ISUDs (n=659), including cocaine use disorder, heroin use disorder, amphetamine or stimulant use disorder, hallucinogen use disorder, club drug use disorder, prescription opioid use disorder, sedative use disorder, or other unspecified drug use disorder. Adults who met criteria for more than one group were classified in the more illegal group.” |
| Data sources/ measurement | 8* | For each variable of interest, give sources of data and details of methods of assessment (measurement). Describe comparability of assessment methods if there is more than one group | 5 | “We used restricted data from the National Epidemiologic Survey on Alcohol and Related Conditions-III (NESARC-III), sponsored by the National Institute on Alcohol Abuse and Alcoholism (NIAAA) (33).” |
| Bias | 9 | Describe any efforts to address potential sources of bias | 15 | “First, the most recent available NESARC survey was conducted between 2012 and 2013, and substance use habits, attitudes and socio-economic conditions of the United States population have shifted since this time.” |
| Study size | 10 | Explain how the study size was arrived at | 9 | “Overall, 16.0% (n= 5,808) of the entire study sample, representing 36,887,003 adults, met criteria for an included SUD within the past 12 months: 659 (11.4%) with ISUDs, representing 4.1 million adults, 828 (14.3%) with CUD, representing 5.1 million adults, and 4,321 with AUD (74.4%), representing 27.7 million adults (Table 1, rows 2-3).” |

Continued on next page

| Quantitative variables | 11 | Explain how quantitative variables were handled in the analyses. If applicable, describe which groupings were chosen and why | 7 | “2.3 Analysis… Comparisons of categorical variables were based on bivariable logistic regression analyses with odds ratios as indicators of effect sizes while continuous variables were compared with Cohen’s d and associated P-values as indicators of statistical significance and effect size differences.” |
| --- | --- | --- | --- | --- |
| Statistical methods | 12 | (*a*) Describe all statistical methods, including those used to control for confounding | 7 | “We constructed three sets of models for each group. The first model was a bivariable logistic regression assessing the unadjusted association MHRQOL with likelihood of SUD treatment. The second model added covariates representing sociodemographic variables such as age, sex, and race that may confound this association. The third model added additional covariates representing behavioral factors such as trouble with police in the past year and diagnostic factors like number of diagnosed non-SUD psychiatric disorders.” |
|  |  | (*b*) Describe any methods used to examine subgroups and interactions | 7 | “First, we investigated differences in sociodemographic, behavioral, and diagnostic characteristics for our three categories of SUDs (ISUD, CUD, and AUD), through three sets of pairwise comparisons (ISUD vs CUD, CUD vs AUD, and ISUD vs AUD).” |
|  |  | (*c*) Explain how missing data were addressed | 7 | “We constructed three sets of models for each group. The first model was a bivariable logistic regression assessing the unadjusted association MHRQOL with likelihood of SUD treatment. The second model added covariates representing sociodemographic variables such as age, sex, and race that may confound this association. The third model added additional covariates representing behavioral factors such as trouble with police in the past year and diagnostic factors like number of diagnosed non-SUD psychiatric disorders.” |
|  |  | (*d*) *Cohort study*—If applicable, explain how loss to follow-up was addressed  *Case-control study*—If applicable, explain how matching of cases and controls was addressed  *Cross-sectional study*—If applicable, describe analytical methods taking account of sampling strategy | 8 | “We used Stata version 17.0 for all analyses and employed the svy commands in Stata to account for the complex survey sampling design of the NESARC-III (e.g., unequal probability of selection, clustering and stratification) (44).” |
|  |  | (*e*) Describe any sensitivity analyses | 8 | “As we aimed to control for three sets of factors in nine multivariable analyses, we undertook a Bonferroni correction for multiple comparisons by considering test results significant at p<0.05/9 = 0.005.” |
| Results | | | | |
| Participants | 13* | (a) Report numbers of individuals at each stage of study—eg numbers potentially eligible, examined for eligibility, confirmed eligible, included in the study, completing follow-up, and analysed | 9 | “3.1 Sample  Overall, 16.0% (n= 5,808) of the entire study sample, representing 36,887,003 adults, met criteria for an included SUD within the past 12 months: 659 (11.4%) with ISUDs, representing 4.1 million adults, 828 (14.3%) with CUD, representing 5.1 million adults, and 4,321 with AUD (74.4%), representing 27.7 million adults (Table 1, rows 2-3).” |
|  |  | (b) Give reasons for non-participation at each stage | N/A | N/A |
|  |  | (c) Consider use of a flow diagram | N/A | N/A |
| Descriptive data | 14* | (a) Give characteristics of study participants (eg demographic, clinical, social) and information on exposures and potential confounders | 9 | “3.2 SUD Legality-Stratified Differences in Background Characteristics and Comorbidities  There were significant differences between pairs of substance groups on many sociodemographic, psychiatric, medical, and behavioral measures (Table 1).” |
|  |  | (b) Indicate number of participants with missing data for each variable of interest | N/A | N/A |
|  |  | (c) *Cohort study*—Summarise follow-up time (eg, average and total amount) | N/A | N/A |
| Outcome data | 15* | *Cohort study*—Report numbers of outcome events or summary measures over time | N/A | N/A |
|  |  | *Case-control study—*Report numbers in each exposure category, or summary measures of exposure | N/A | N/A |
|  |  | *Cross-sectional study—*Report numbers of outcome events or summary measures | 10 | “Overall, 627 patients (10.7%), representing 3.8 million adults, were treated for SUDs in the past 12 months, while 5,181 patients (89.2%), representing 33.1 million adults, did not receive treatment. Treatment rates varied significantly by legality class of substances of abuse (Table 2): 26.9% of those with past-year ISUDs, representing 1.1 million US adults, received SUD treatment, while just 10.3% of those with past-year CUD (representing 612,389 US adults) and 8.5% of those with past-year AUD (representing 2.1 million US adults) received treatment.” |
| Main results | 16 | (*a*) Give unadjusted estimates and, if applicable, confounder-adjusted estimates and their precision (eg, 95% confidence interval). Make clear which confounders were adjusted for and why they were included | 11 | “3.5 Multivariate Group Comparisons on Measures of MHRQOL  Multivariable-adjusted comparisons within legality-based groups of likelihood of seeking treatment focused on the independent contribution (or lack thereof) of MHRQOL to the likelihood of seeking treatment net of other factors. Unadjusted regression coefficients are presented in columns 2, 5 and 8 of Table 4 and reflect differences in MHRQOL between in treated and non-treated subgroups within each legality group. In unadjusted bivariable logistic regression, higher MHRQOL is significantly and negatively associated with likelihood of treatment in those with CUD (-4.1% for each additional point of MCS) and AUD (-5.0% for each additional point of MCS), but there was no significant association among those with ISUD (Table 4, Columns 2, 5 and 8).” |
|  |  | (*b*) Report category boundaries when continuous variables were categorized | 11 | See Table 4 |
|  |  | (*c*) If relevant, consider translating estimates of relative risk into absolute risk for a meaningful time period | N/A | N/A |

Continued on next page

| Other analyses | 17 | Report other analyses done—eg analyses of subgroups and interactions, and sensitivity analyses | 11 | “3.5 Multivariate Group Comparisons on Measures of MHRQOL  Multivariable-adjusted comparisons within legality-based groups of likelihood of seeking treatment focused on the independent contribution (or lack thereof) of MHRQOL to the likelihood of seeking treatment net of other factors. Unadjusted regression coefficients are presented in columns 2, 5 and 8 of Table 4 and reflect differences in MHRQOL between in treated and non-treated subgroups within each legality group. In unadjusted bivariable logistic regression, higher MHRQOL is significantly and negatively associated with likelihood of treatment in those with CUD (-4.1% for each additional point of MCS) and AUD (-5.0% for each additional point of MCS), but there was no significant association among those with ISUD (Table 4, Columns 2, 5 and 8)…” |
| --- | --- | --- | --- | --- |
| Discussion | | | | |
| Key results | 18 | Summarise key results with reference to study objectives | 13 | “The results revealed, first, significantly greater likelihood of service use among those with ISUDs as compared to partially legal CUD and fully legal AUD and second, significant differences in sociodemographic and clinical characteristics among groups classified by legal status of the substances underlying their SUDs. Third, the results revealed further differences between these groups in individual characteristics associated with treatment use, especially a significant association between lower MHRQOL and treatment use among individuals with the partially legal CUD and entirely legal AUD. In contrast, no significant association was observed between lower MHRQOL and service use among those with ISUDs.” |
| Limitations | 19 | Discuss limitations of the study, taking into account sources of potential bias or imprecision. Discuss both direction and magnitude of any potential bias | 15 | “Several limitations of this study deserve comment. First, the most recent available NESARC survey was conducted between 2012 and 2013, and substance use habits, attitudes and socio-economic conditions of the United States population have shifted since this time.” |
| Interpretation | 20 | Give a cautious overall interpretation of results considering objectives, limitations, multiplicity of analyses, results from similar studies, and other relevant evidence | 16 | “Despite these limitations, to our knowledge this is the first study to characterize the differences in proportions and correlates of adults receiving treatment for SUD by the legal status of the substances involved…” |
| Generalisability | 21 | Discuss the generalisability (external validity) of the study results | 16 | “As legalization progresses, if indeed it does, there may be less latent legal pressure for treatment seeking and a greater need for effective methods of public education on the long-term adverse effects of SUDS to motivate use of treatment, as well as for harm reduction interventions for those who make hazardous, if legal, use of harmful substances.” |
| Other information | |  | | |
| Funding | 22 | Give the source of funding and the role of the funders for the present study and, if applicable, for the original study on which the present article is based | N/A | N/A |

*Give information separately for cases and controls in case-control studies and, if applicable, for exposed and unexposed groups in cohort and cross-sectional studies.

**Note:** An Explanation and Elaboration article discusses each checklist item and gives methodological background and published examples of transparent reporting. The STROBE checklist is best used in conjunction with this article (freely available on the Web sites of PLoS Medicine at http://www.plosmedicine.org/, Annals of Internal Medicine at http://www.annals.org/, and Epidemiology at http://www.epidem.com/). Information on the STROBE Initiative is available at www.strobe-statement.org.
